# Supplementary material for: Fluorene-9-bisphenol is anti-oestrogenic and may cause adverse pregnancy outcomes in mice
Source: Nat Commun. 2017 Mar 1;8:14585. doi: 10.1038/ncomms14585 (PMC5339874; doi:10.1038/ncomms14585)
Supplement: Supplementary Information — Supplementary figures, supplementary tables and supplementary notes. [file ncomms14585-s1.pdf]

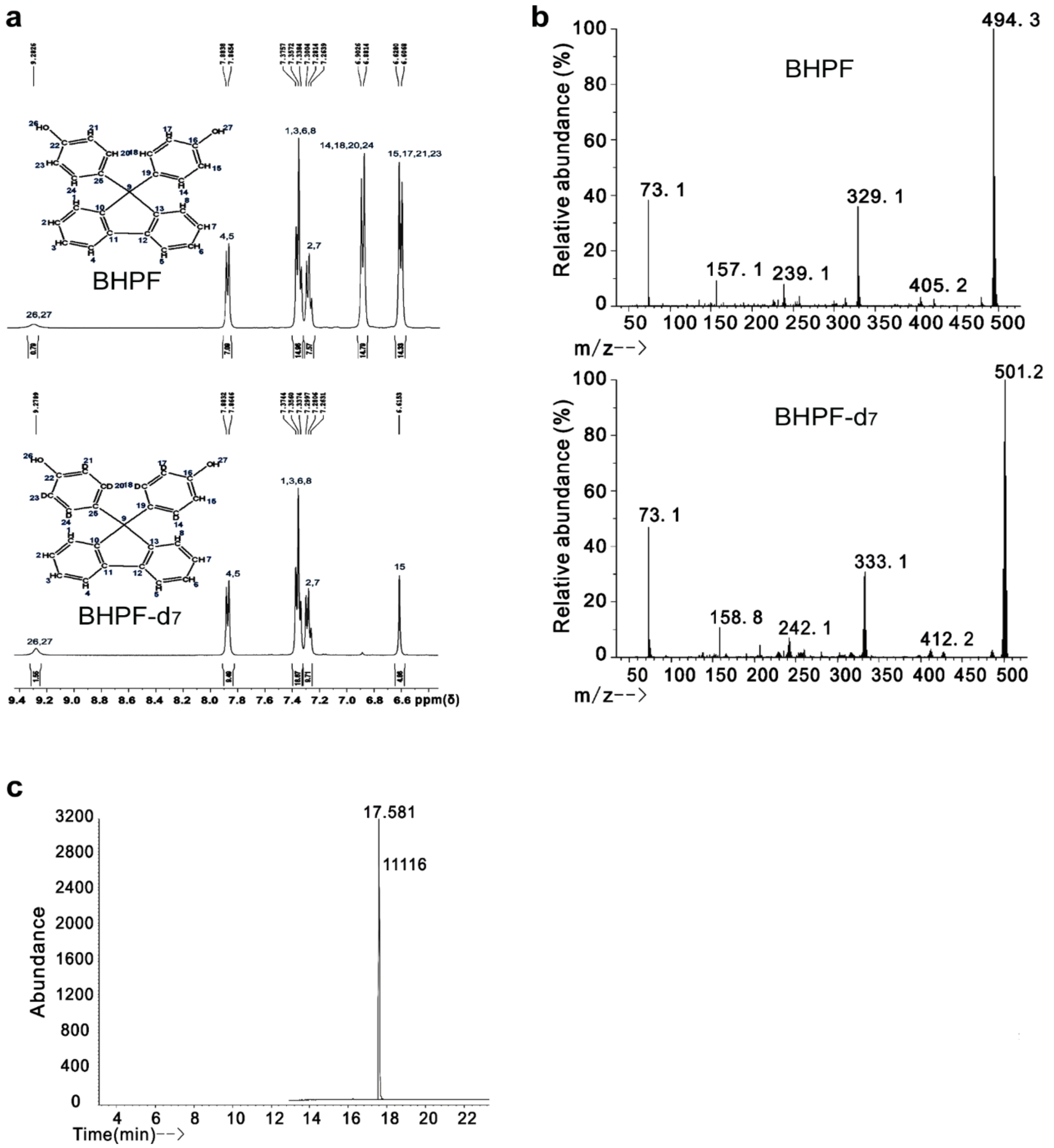

Supplementary Figure 1. Method development for determination of BHPF in water samples by gas chromatography-mass spectroscopy (GC-MS). **(a)** 1H NMR spectra of BHPF (up) and the synthesized deuterated internal standard of BHPF (BHPF-d7) (down): 1H NMR (400MHz, DMSO-d6, TMS, ppm). δ; 9.27~9.29 (s, 2H, H26, H27), 7.86~7.88 (d, 2H, H4, H5), 7.26~7.38 (m, 6H, H1, H2, H3, H6, H7, H8), 6.88 ~ 6. 90 (d, 4H, H14, H18, H20, H24), 6.60 ~ 6.63 (d, 4H, H15, H17, H21, H23); **(b)** The mass spectra of O-bis(trimethylsilyl) derivatives of BHPF and BHPF-d7 at retention time of 17.581 min; **(c)** A typical GC-MS chromatogram of O-bis(trimethylsilyl) derivative of BHPF extracted from water that was filled in a PC plastic drink bottle.

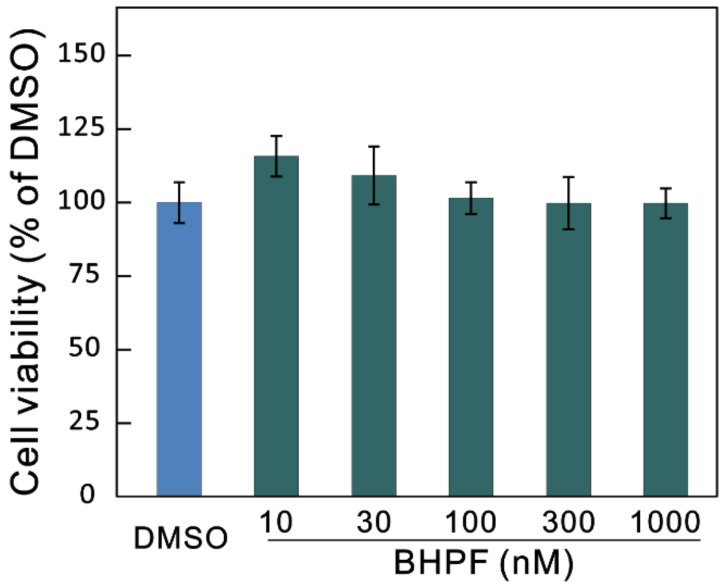

Supplementary Figure 2. JEG-3 cell viability results after BHPF treatment. The data were expressed as percentage of control. The assay was performed in quadruplicate and the data were expressed as mean ± standard deviation.

Supplementary Table 1: Concentrations (ng/L) of BHPF in the Drinking water samples that was filled in plastic bottles hotly.

Bottle types	Materials [a]	N	Mean ± s.d.	Minimum	Maximum	Detection (%)
adult bottle	PC	18	19.51 ± 29.55	ND	81.47	61.11
	Tritan	1	9.49	--	9.49	100
	PP	3	ND	ND	--	0
Children bottles and sippy cups	PC	12	10.17±7.53	ND	20.51	58.33
	Tritan	1	12.87	--	12.87	100
	PP	2	ND	ND	--	0
Baby milk bottles	PC	3	5.61±3.95	2.01	9.73	100
	PA	1	ND	ND	--	0
	PPSU	6	ND	ND	--	0
	PP	5	ND	ND	--	0

PC, Polycarbonates; Tritan, Eastman Tritan^{TM} copolyester; PP, Polypropylene; PA, Polyamide; PPSU, Polyethersulfone; ND, not detected.

Supplementary Table 2: Selected gene expression changes validated by quantitative real-time RT-PCR. GAPDH was used as endogenous control.

gene	liver		ovary		uterus	
symbol	Q-RT-PCR	Microarray	Q-RT-PCR	Microarray	Q-RT-PCR	Microarray
Spp1	-1.51	-1.30	-1.68	-1.67	3.43	2.57
Cfd	-2.43	-3.11	2.28	4.13	-2.85	-6.54
Orm1	-1.41	-1.65	2.19	1.18	5.17	23.12
Sprr2f	4.41	3.69	-5.28	-5.81	-5.78	-2.52
Sprr2b	4.76	2.75	-9.65	-2.07	-9.58	-13.20
Sprr2a2	3.43	2.10	-8.94	-12.01	-5.70	-5.24
Sprr2g	3.68	2.09	-5.70	-5.71	-1.83	-2.13
Sprr1a	4.11	2.17	1.16	1.17	-2.01	-3.44
Il1f6	2.66	1.95	-4.29	-1.11	-4.66	-10.96
serp1d	2.48	1.02	1.59	1.13	30.91	17.78
cyp2e1	4.06	-1.01	1.69	1.25	4.00	5.02
chil3	-2.81	-8.53	-3.92	-9.16	-3.07	-6.60
GAPDH	1.00	1.02	1.00	1.03	1.00	-1.02
Actb	1.25	1.09	1.13	1.34	1.13	1.07

Supplementary Table 3: Primers and procedures of RT-PCR for amplifying of human estrogen receptor α (*ERα*) and human estrogen receptor β (*ERβ*).

Gene (Accession No.)		Primer sequences (5' to 3')	PCR condition			
			Denaturation	Annealing	Elongation	Cycle No.
ERα	Forward	ACGGACCATGACCATGACCC	98 °C	58 °C	68 °C	35
(NM_000125)	Reverse	TCTCAGACCGTGGCAGGGAA	10 sec	30 sec	120 sec	
ERβ	Forward	TCTCAGCTGTTATCTCAAGACATGG	98 °C	55 °C	68 °C	35
(NM_001437)	Reverse	CGTCACTGAGACTGTGGGTTC	10 sec	30 sec	120 sec	

Supplementary Table 4: Primers of Selected Genes for Quantitative Real-Time PCR.

Gene Symbol	Accession Number	Sequence of Forward Primer (5' to 3')	Sequence of Reverse Primer (5' to 3')
Spp1	NM_001204233	GAGAGCGAGGATTCTGTGGA	CTTGTGTACTAGCAGTGACGGTC
Cfd	NM_013459	GCTATCCCAGAATGCCTCGTT	GGTTCCACTTCTTTGTCCTCGTA
Orm1	NM_008768	CACCAAACCCCACCCCT	GATATTGACTATGTCAGCAAACCTT
Sprr2f	NM_011472	ATGGGTCTTGTTCCATTGTTCA	AACAGTAACAACTACCCTGCTCAAG
Sprr2b	NM_011469	GTGTCCACCCAAGAATAAATGAG	AGGACAGGCGTTCAAAGGAG
Sprr1a	NM_009264	CTTAACATTGCCCAAAGTCATA	CACTTGTCTCACTCCTCACCC
Sprr2a2	NM_001164787	GGTCACTGCTGTTTCATTTCCT	ATTAGACCATCACCAAAGGGG
Sprr2g	NR_003548	CTAGTAGATGTCCCTCAGTGCCTT	AGCAAATGGAACATCCGTGA
ll1f6	NM_019450	AGAAGCAATCAAAGACACCCCTA	GCATCTGATTCACCACCAAGG
serp1d	NM_009246	GAAAGATAGTTGAGGCGGTGAA	TCTCAGGATCGAATGGCTGTT
cyp2e1	NM_021282	CACCCTCCTCCTCGTATCCAT	AAAGAAAGGAATTGGGAAAGGTC
chil3	NM_009892	GGAGGATGGAAGTTTGGA	TCTGACGGTTCTGAGGAGTA
GAPDH	NM_008084	TGCCCCCATGTTTGTGATG	TGTGGTCATGAGCCCTTCC
Actb	NM_007393	GCCCTGAGGCTCTTTTCCAG	TGCCACAGGATTCCATACCC

Supplementary Table 5. The organ parameters of female mice at the age of PND 60 in the subchronic and reproductive Toxicity tests.

Endpoints	Ctrl	0.4 mg BHPF/kg	2 mg BHPF/kg	10 mg BHPF/kg	50 mg BHPF/kg	1.2 mg TAM/kg
Relative liver weight (%)	4.66 ± 0.30	5.25 ± 0.46	4.90 ± 0.35	4.72 ± 0.37	4.58 ± 1.46	4.24 ± 0.23
Relative kidney weight (%)	0.66 ± 0.07	0.68 ± 0.07	0.65 ± 0.04	0.67 ± 0.03	0.64 ± 0.09	0.63 ± 0.09
Relative spleen weight (%)	0.44 ± 0.06	0.42 ± 0.07	0.45 ± 0.08	0.43 ± 0.08	0.49 ± 0.11	0.40 ± 0.06
Relative uterine weight (%)	0.59 ± 0.13	0.47 ± 0.15	0.44 ± 0.18*	0.40 ± 0.10**	0.38 ± 0.06**	0.45 ± 0.16*
Relative weight of the left ovary (%)	0.028 ± 0.005	0.030 ± 0.007	0.029 ± 0.005	0.031 ± 0.008	0.028 ± 0.008	0.018 ± 0.004**
Relative weight of the right ovary (%)	0.030 ± 0.007	0.034 ± 0.009	0.030 ± 0.007	0.030 ± 0.007	0.030 ± 0.007	0.020 ± 0.003**

Relative organ weights are presented as the percentage of organ weight to body weight. Data are expressed as mean ± standard deviation. Ten mice per group were used for the test. ** $P < 0.01$ compared with corresponding control. Significance was tested by Fisher's LSD test.

Supplementary Table 6. The organ parameters of male mice at the age of PND 84 in the subchronic and reproductive Toxicity tests.

Endpoints	Ctrl	0.4 mg BHPF/kg	2 mg BHPF/kg	10 mg BHPF/kg	50 mg BHPF/kg	1.2 mg TAM/kg
Relative liver weight (%)	4.76 ± 0.51	5.06 ± 0.31	5.07 ± 0.60	4.85 ± 0.34	5.01 ± 0.41	5.09 ± 0.57
Relative kidney weight (%)	0.796 ± 0.095	0.841 ± 0.078	0.849 ± 0.081	0.803 ± 0.085	0.834 ± 0.122	0.775 ± 0.066
Relative spleen weight (%)	0.281 ± 0.057	0.298 ± 0.049	0.288 ± 0.047	0.295 ± 0.049	0.266 ± 0.049	0.312 ± 0.069
Relative testis weight (%)	0.335 ± 0.049	0.353 ± 0.048	0.334 ± 0.039	0.353 ± 0.054	0.323 ± 0.025	0.319 ± 0.044
Relative weight of seminal vesicle (%)	0.86 ± 0.12	0.92 ± 0.17	0.85 ± 0.12	0.97 ± 0.14*	0.86 ± 0.16	0.71 ± 0.16
Relative prostate weight (%)	0.090 ± 0.016	0.093 ± 0.016	0.093 ± 0.016	0.100 ± 0.019	0.087 ± 0.020	0.096 ± 0.020
Relative epididymis weight (%)	0.119 ± 0.012	0.119 ± 0.011	0.121 ± 0.006	0.126 ± 0.011*	0.117 ± 0.009	0.116 ± 0.011

Relative organ weights are presented as the percentage of organ weight to body weight. Data are expressed as mean ± standard deviation. Fifteen mice per group were used for the test. * $P < 0.05$ compared with corresponding control; ** $P < 0.01$ compared with corresponding control. Significance was tested by Fisher's LSD test.

Supplementary Table 7. The organ parameters of dams in the subchronic and reproductive Toxicity tests.

Endpoints	Ctrl	0.4 mg BHPF/kg	2 mg BHPF/kg	10 mg BHPF/kg	50 mg BHPF/kg
Relative liver weight (%)	6.69 ± 0.40	6.72 ± 0.55	6.91 ± 0.64	6.78 ± 0.28	6.71 ± 0.31
Relative kidney weight (%)	0.58 ± 0.07	0.56 ± 0.05	0.55 ± 0.05	0.56 ± 0.05	0.57 ± 0.04
Relative spleen weight (%)	0.64 ± 0.23	0.74 ± 0.41	0.75 ± 0.44	0.75 ± 0.29	0.50 ± 0.11
Relative weight of the left ovary (%)	0.022 ± 0.005	0.021 ± 0.004	0.020 ± 0.003	0.021 ± 0.004	0.021 ± 0.005
Relative weight of the right ovary (%)	0.024 ± 0.005	0.022 ± 0.004	0.024 ± 0.002	0.023 ± 0.003	0.024 ± 0.004

Relative organ weights are presented as the percentage of organ weight to body weight. Data are expressed as mean ± standard deviation. Fifteen mice per group were used for the test. * $P < 0.05$ compared with corresponding control; ** $P < 0.01$ compared with corresponding control. Significance was tested by Fisher's LSD test.

Supplementary Note 1: Microarray data analyses

Analyses of global gene expression profiles in the livers, ovaries, and uteri of mice treated with 50 mg/kg bw/d of BHPF for 3 days compared with those of control mice was performed by means of an Agilent mouse gene expression microarray (Agilent Technologies). In the livers, the expressions of 2963 of the 55,821 genes and expressed-sequence tags (ESTs) evaluated was modified by at least two fold in mice after BHPF treatment (up-regulated in 77.7% and down-regulated in 22.3%). GO analysis showed that 1363 genes were enriched in 207 biological process categories, such as defense responses, responses to stimuli, responses to other organisms, multi-organism processes, immune system processes, responses to biotic stimuli, and inflammatory responses. Pathway enrichment analyses showed that 302 altered genes in the liver were enriched in 29 pathways, such as natural killer cell–mediated cytotoxicity, hematopoietic cell lineage, and autoimmune thyroid disease. In the ovaries, the expressions of 948 genes and ESTs was up-regulated (34.5%) or down-regulated (65.5%) by at least two fold after BHPF treatment. Among them, 488 genes were enriched in 45 biological process categories, including immune system processes, responses to stimuli, responses to E_2 stimuli, responses to chemical stimuli, and immune responses. The genes associated with 'response to E_2 stimuli' were *sprr2a*, *sprr2b*, *sprr2f*, and *sprr2g*, which were down-regulated by BHPF. Pathway analyses showed that 98 genes were enriched in 14 pathways, including natural killer cell–mediated cytotoxicity, viral myocarditis, autoimmune thyroid disease, and the B-cell receptor-signaling pathway. In the uteri, the expressions of 1324 genes or ESTs was up-regulated (56.6%) or down-regulated (43.4%) by at least twofold after BHPF treatment. Of them, 534 genes were enriched in 200 biological processes, including responses to stimuli, responses to chemical stimuli, responses to E_2 stimuli, oxidation-reduction processes, cholesterol homeostasis and efflux, regulation of cholesterol absorption, acute-phase responses, and steroid metabolic processes. Pathway analyses showed that 228 genes were enriched in 27 pathways, including complement and coagulation cascades, drug metabolism, retinol metabolism, metabolism of xenobiotics by cytochrome P450, steroid hormone biosynthesis, and androgen and estrogen metabolism.

Supplementary Note 2: WST-1 cell proliferation assay

Cell viability was determined using the cell proliferation reagent WST-1 (DOJINDO, Mashiki, Japan). Aliquots (200 μL) of JEG-3 cells (1×10^4 cells/well) were seeded into 96 well-plates and precultured for 24 h. The cells were then treated with various concentrations of BHPF. After another 24 hours, 20 μL of 5 mM WST-1 with 0.2 mM 1-Methoxy-5-methylphenazinium methylsulfate was added for four hours before the absorbance at 490 nm was measured with a microplate reader (iMark, BioRad). The assay was performed in quadruplicate and results were expressed as mean ± standard deviation.

Supplementary Note 3: Supplementary description of animal experiments

Immature female CD-1 mice were obtained from Experimental Animal Tech Co. of Weitonglihua (Beijing, China). Mice with a maximal difference in body weight of 1 g were selected for the experiments and randomly assigned to either the treatment or control groups. For the oral gavage treatment experiments, the mice in each cage were labeled by shaving hair on different parts of the body, and the oral gavage treatment was performed in turns for each group, whereby in each turn only one mouse in a group was treated. After the period of treatment, the mice were weighed and sacrificed 24 h after the final treatment according to the sequence of the treatments, and the uteri were removed, blotted, and weighed by one experimenter to reduce the risk of bias in the data collection. For the drinking water treatment experiments, the mice were weighed and sacrificed in turns for each group, whereby in each turn only one mouse in a group was sacrificed; the uteri were removed, blotted, and weighed by one experimenter to reduce the risk of bias in the data collection.
